# Supplementary material for: Complexome profiling of the Chlamydomonas psb28 mutant reveals TEF5 as an early PSII assembly factor
Source: Plant Cell. 2025 Mar 17;37(6):koaf055. doi: 10.1093/plcell/koaf055 (PMC12164590; doi:10.1093/plcell/koaf055)
Supplement: koaf055_Supplementary_Data [file koaf055_supplementary_data.zip › psb28_interactive.zip!/plots/intensity/qualityPlots/peptides/MPPB_Cre12.g523850.t1.2.html]

### Cre12.g523850.t1.2

Trivial name: MPPB  
SeqCoverage [median]: 51.50  
Mass: 55.05 kDa  
  
 

| Sequence | MissedCleavage | Mass [Da] | wtIntensity | muIntensity | Score |
| --- | --- | --- | --- | --- | --- |
| AEMFAR | 0 | 723.34 | 6253.03 | 22159.75 | 137.50 |
| AIDKERDVILR | 2 | 1326.76 | 0.00 | 2991.20 | 57.27 |
| ASLMFFQDSTHHVAESIGR | 0 | 2132.01 | 85963.07 | 125663.91 | 176.11 |
| AVNILSDILLNSNLDAR | 0 | 1840.01 | 20331.45 | 0.00 | 266.97 |
| DQLVEYMK | 0 | 1024.49 | 16370.57 | 12101.92 | 117.40 |
| DRSEDFAYAIMSNLTR | 1 | 1887.88 | 3427.37 | 2149.86 | 66.97 |
| DVVATDANPFLR | 0 | 1316.67 | 92781.45 | 120339.30 | 245.50 |
| ELEVEVENMGGQLNAYTGR | 0 | 2107.98 | 6356.26 | 11604.31 | 156.13 |
| ELLVYGR | 0 | 848.48 | 88275.92 | 103763.50 | 150.07 |
| EMEEVNK | 0 | 877.39 | 0.00 | 1407.11 | 119.56 |
| EMEEVNKQTSELVFDHLHATAFQYSPLGR | 1 | 3375.62 | 8700.67 | 0.00 | 79.88 |
| FSNPRPSPIDHTPLLSTLPETR | 1 | 2474.29 | 95894.85 | 82361.93 | 125.04 |
| FTGSYVHDR | 0 | 1080.50 | 3692.64 | 3293.20 | 71.45 |
| IDAVDANAIR | 0 | 1056.56 | 157413.34 | 176858.28 | 226.64 |
| ITTLPNGLR | 0 | 983.58 | 4861.06 | 31829.09 | 145.04 |
| LASDAFGSVPDEDAATSVR | 0 | 1906.89 | 69461.68 | 137268.63 | 225.13 |
| MVLAAAGAVNHDELVK | 0 | 1636.86 | 109854.60 | 162814.05 | 167.55 |
| PSPIDHTPLLSTLPETR | 0 | 1872.99 | 2000.51 | 0.00 | 100.41 |
| QTSELVFDHLHATAFQYSPLGR | 0 | 2516.24 | 5734.18 | 0.00 | 89.87 |
| SEDFAYAIMSNLTR | 0 | 1616.75 | 7764.73 | 10986.83 | 118.48 |
| SINRDQLVEYMK | 1 | 1494.75 | 21104.23 | 20797.80 | 131.26 |
| SLLVKEPSR | 1 | 1027.60 | 2777.70 | 13290.75 | 87.31 |
| SVKELEVEVENMGGQLNAYTGR | 1 | 2422.18 | 5544.27 | 5311.54 | 121.25 |
| TILGPVENIK | 0 | 1082.63 | 103237.37 | 198381.27 | 152.86 |
|  |  |  | 917800.9 | 1245374.2 |  |
